# Role of preoperative magnetic resonance imaging and histological assessment in identifying patients with a low risk of endometrial cancer: a Korean Gynecologic Oncology Group ancillary study

**DOI:** 10.18632/oncotarget.22520

**Published:** 2017-11-20

**Authors:** Jung-Yun Lee, Yun Hwan Kim, Jong-Min Lee, Kidong Kim, Sokbom Kang, Myong Cheol Lim, Beob-Jong Kim, Bang Hyun Lee, Jae Weon Kim

**Affiliations:** ^1^ Department of Obstetrics and Gynecology, Institute of Women's Life Medical Science, Yonsei University College of Medicine, Seoul, Korea; ^2^ Department of Obstetrics and Gynecology, Ehwa University College of Medicine, Seoul, Korea; ^3^ Department of Obstetrics and Gynecology, Kyung Hee University College of Medicine, Seoul, Korea; ^4^ Department of Obstetrics and Gynecology, Seoul National University Bundang Hospital, Seongnam, Korea; ^5^ Gynecologic Oncology Research Branch, Research Institute and Hospital, and Department of Cancer Control and Public Health, Graduate School of Cancer Science and Policy, National Cancer Center, Goyang, Korea; ^6^ Department of Obstetrics and Gynecology, Korea Cancer Hospital, Korea Cancer Institute of Radiological and Medical Sciences, Seoul, Korea; ^7^ Department of Obstetrics and Gynecology, Hallym University Kangdong Sacred Heart Hospital, Seoul, Korea; ^8^ Department of Obstetrics and Gynecology, Seoul National University College of Medicine, Seoul, Korea

**Keywords:** endometrial cancer, guideline, low risk, lymphadenectomy, magnetic resonance imaging

## Abstract

Preoperative identification of individuals at low risk of lymph node metastasis is key to the proper management of endometrial cancer. This study evaluated the role of preoperative assessment based on magnetic resonance imaging (MRI) and histological analysis in identifying a group having a low risk of lymph node metastasis. Data of 529 patients with endometrial cancer were obtained from a prospective multicenter database, between January 2012 and December 2014. Clinical staging, based on MRI and histological analysis, was compared with final pathology results after the surgical staging procedure. The preoperative low-risk criteria, based on current guidelines from Korea, France, and Canada, and criteria used for fertility-sparing therapies, were applied to our multicenter cohort and the accuracy of each set of criteria for identifying group at low risk of lymph node metastasis was evaluated. When considering grades or MR stages separately, the overall agreement between preoperative and postoperative findings was poor (Kappa 0.45 for grades; 0.41 for stages). However, when combining these two parameters, the low-risk group, as defined by any of the guidelines, had an acceptable rate of lymph node metastasis (below 3%). The French guidelines identified 249 patients (47.1%) as being in the low-risk group. Criteria used to define fertility-sparing therapy candidates identified 48 patients (9.1%) among the study population, only one of whom had extra-uterine disease. This study shows that the current guidelines, using preoperative assessment based on MRI and histological analysis, can identify low-risk patients, who may be candidates for omitting lymphadenectomy.

## INTRODUCTION

In 1988, the International Federation of Obstetrics and Gynecology (FIGO) recommended surgical staging for endometrial cancer patients [1]. Although two randomized controlled studies failed to show a survival benefit for performing lymphadenectomy over not performing this procedure, the value of lymphadenectomy for the treatment of endometrial cancer remains controversial [2, 3]. The current guidelines recommend that lymphadenectomy should be performed for selected patients, but the need for lymphadenectomy could be avoided in a low-risk group [4–8]. Therefore, preoperative and intraoperative assessment is key to the proper surgical management of this disease subset.

Recently, the Korean Gynecologic Oncology Group (KGOG) proposed a preoperative prediction model for lymph node metastasis, using serum CA-125 levels and MRI parameters [9]. In addition, the accuracy and reliability of this model for identifying a low-risk group has been confirmed in a prospective cohort [10]. However, approximately 60% of KGOG members prefer to use routine lymphadenectomy, even in patients satisfying the KGOG-2015 low-risk criteria [11], and some decide on lymphadenectomy based on preoperative imaging and the grade of the tumor, as determined through biopsies, prior to surgery.

Each guideline proposes specific criteria for the low-risk group, based on various combinations of preoperative histology and MRI parameters. In addition, decisions about fertility-sparing therapies are solely dependent on clinical staging, based on preoperative assessment [4, 12]. However, the actual risk of extra-uterine disease in patients that satisfy the above-mentioned criteria has not yet been determined in diverse clinical settings.

The aim of this study was to evaluate the rate of upgrading and upstaging between preoperative assessment and final surgical pathology in a multicenter prospective cohort of patients with endometrial cancer who had undergone surgical staging (KGOG-2015) [10]. In addition, we evaluated the performance of preoperative assessment based on current guidelines and fertility-sparing therapies for identifying a group at low risk of endometrial cancer.

## RESULTS

### Comparison of clinical and surgical staging

A total of 529 patients were included in the analysis (Table 1). The overall agreement between preoperative and postoperative grading was poor, according to the Kappa statistics (0.45). Among the 244 cases with preoperative grade 1 disease, the preoperative and postoperative pathology were in agreement in 190 (77.9%) patients, but the final pathology grade was advanced to grade 2 in 41 (16.8%) and to grade 3 in 7 (2.9%) of these patients (Table 2). Among the 127 cases with preoperative grade 2 disease, 72 (56.7%) showed correlation with the final pathology report, whereas 17 (13.4%) were upgraded to grade 3.

**Table 1:** Characteristics of study population

	No. (%)
Age, median (range), y	53 (28-81)
Menopause	348 (65.8)
Route of surgery	
Laparotomy	102 (19.3)
Laparoscopy/robotic	427 (80.7)
Method of biopsy	
Dilatation and curettage	427 (80.7)
Other	102 (19.3)
Preoperative serum CA-125, median (range), U/mL	17.7 (1.0-1138.3)
Histologic type	
Endometrioid	465 (87.9)
Nonendometrioid	64 (12.1)
Tumor grade	
I/II	416 (78.6)
III or not determined	113 (21.4)
Myometrial invasion	
<1/2	406 (76.7)
≥1/2	123 (23.3)
Tumor size	
<2cm	229 (43.3)
≥2cm	300 (56.7)
Lymphovascular space invasion	
No	431 (81.5)
Yes	98 (18.5)
Para-aortic lymph node dissection	
No	305 (57.7)
Yes	224 (42.3)

**Table 2:** Correlation between preoperative assessment and final pathology

a) Grade			
Postoperative	Preoperative		
	G1	G2	G3
G1	190 (77.9%)	36 (28.4%)	4 (6.8%)
G2	41 (16.8%)	72 (56.7%)	26 (26.3%)
G3	7 (2.9%)	17 (13.4%)	12 (12.1%)
N/A	6 (2.5%)	2 (1.6%)	24 (24.2%)
Upgraded patients (%)	19.7	13.4	N/A

**Table:** 

b) Stage							
Pathologic stage	Preoperative stage by magnetic resonance imaging						
	IA	IB	II	IIIA	IIIB	IIIC	IV
IA	305 (86.2%)	24 (35.8%)	5 (21.7%)	3 (100%)	0 (0%)	24 (34.3%)	5 (41.7%)
IB	22 (6.2%)	30 (44.8%)	4 (17.4%)	0 (0%)	0 (0%)	9 (12.9%)	3 (25.0%)
II	12 (3.4%)	5 (7.5%)	9 (39.1%)	0 (0%)	0 (0%)	3 (4.3%)	1 (8.3%)
IIIA	0 (0%)	3 (4.5%)	3 (13.0%)	0 (0%)	0 (0%)	2 (2.9%)	1 (8.3%)
IIIB	0 (0%)	0 (0%)	0 (0%)	0 (0%)	0 (0%)	1 (1.4%)	0 (0%)
IIIC	12 (3.4%)	5 (7.5%)	1 (4.4%)	0 (0%)	0 (0%)	23 (32.9%)	1 (8.3%)
IV	3 (0.9%)	0	1 (4.4%)	0 (0%)	0 (0%)	8 (11.4%)	1 (8.3%)
Upstaged patients (%)	13.8	19.4	21.7	0	0	11.4	0

The performance of preoperative MRI in predicting the final stage is reported in Table 2. Concordance between the preoperative and postoperative MR stage was 67.9%, with a kappa of 0.41. Of the 354 patients with preoperative MRI stage IA, 49 (13.8%) patients were upstaged; 12 (3.4%) patients with lymph node metastasis were categorized as having stage IIIC, and 3 (0.9%) patients with extra-uterine metastasis as having stage IV disease.

### Performance of preoperative low-risk criteria by current guidelines

The performance of the KSGO, SOGC, INCa low-risk criteria, and fertility-sparing therapies was assessed by applying them to all 529 patients (Table 3).

**Table 3:** Evaluation of low-risk criteria according to KSGO, SOGC, INCa, and fertility-sparing therapy candidates

	Low risk group			FSS candidates	
	KSGO (n=178)	SOGC (n=204)	INCa (n=249)	NCCN (n=48)	KGOG 2020 (n=99)
Pathologic stage					
IA	166 (93.3%)	176 (86.3%)	225 (90.4%)	47 (97.9%)	94 (95.0%)
IB	8 (4.5%)	21 (10.3%)	12 (4.8%)	0 (0%)	0 (0%)
II	1 (0.6%)	1 (0.5%)	6 (2.4%)	0 (0%)	3 (3.0%)
IIIA	0 (0%)	2 (1.0%)	0 (0%)	0 (0%)	0 (0%)
IIIB	0 (0%)	0 (0%)	0 (0%)	0 (0%)	0 (0%)
IIIC	3 (1.7%)	4 (2.0%)	6 (2.4%)	1 (2.1%)	2 (2.0%)
IV	0 (0%)	0 (0%)	0 (0%)	0 (0%)	0 (0%)
≥Stage II	4 (2.3%)	7 (3.4%)	12 (4.8%)	1 (2.1%)	5 (5.1%)
Lymph node metastasis	3 (1.7%)	4 (2.0%)	6 (2.4%)	1 (2.1%)	2 (2.0%)
AUC^*^ (95% CI)	0.65 (0.62-0.69)	0.67 (0.63-0.71)	0.70 (0.65-0.75)	0.54 (0.52-0.56)	0.58 (0.55-0.61)

^*^For the diagnosis of lymph node metastasis.

FSS, fertility-sparing surgery; KSGO, Korean Society of Gynecologic Oncology; SOGC, Society of Obstetricians and Gynaecologists of Canada; INCa, French National Cancer Institute (INCa); NCCN, National Cancer Comprehensive Network; KGOG, Korean Gynecologic Oncology Group; AUC, area under the curve; CI, confidence interval.

The KSGO guidelines identified 178 patients as being in the low-risk group for lymph node metastasis. Of these 178 patients, only 3 patients (1.7%) actually had lymph node metastasis. For the diagnosis of lymph node metastasis, the KSGO low-risk criteria had an AUC of 0.65 (95% CI: 0.62-0.69). The SOGC guidelines identified 204 patients with preoperative grade 1 and presumed stage I disease. In this low-risk group, only 4 patients (2.0%) had lymph node metastasis. For the diagnosis of lymph node metastasis, the SOGC low-risk criteria had an AUC of 0.67 (95% CI: 0.63-0.71). The INCa guidelines identified 249 patients as being in the low-risk group for lymph node metastasis. Among these patients in the low-risk group, lymph node metastasis was found only in 6 patients (2.4%). For the diagnosis of lymph node metastasis, the INCa criteria had an AUC of 0.70 (95% CI: 0.65-0.75).

For fertility-sparing options, the NCCN suggests the following low-risk criteria: MR stage IA, endometrioid, grade 1, and young women desiring fertility preservation. In our database, 48 patients were candidates for fertility-sparing therapies. Of the 48 patients, only one patient (2.1%), who had lymph node metastasis, had extra-uterine disease. These criteria had an AUC of 0.54 (95% CI: 0.52-0.56) for the diagnosis of lymph node metastasis. When expanding fertility-sparing therapies to KGOG-2020, 99 patients were identified. Of these, two patients (2.0%) already had lymph node metastasis.

**Figure 1:**
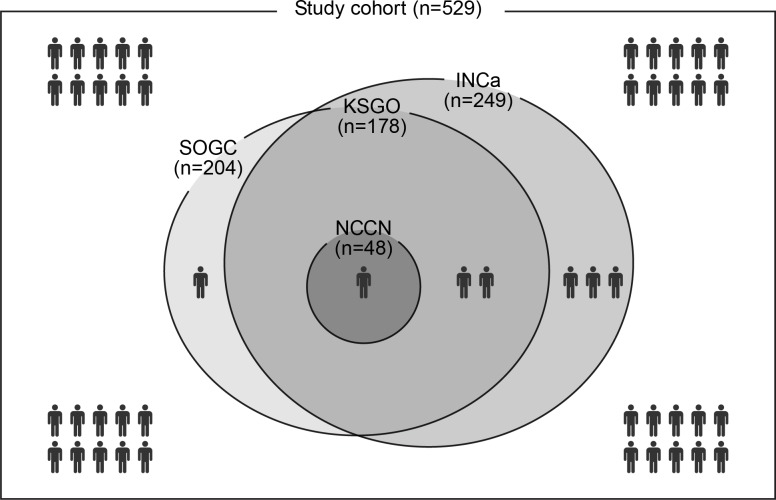
Venn diagram for distribution of low-risk group according to various criteria The area of circle is proportional to the number of low risk group per criteria. Lymph node metastasis is described by patient icon.

We used a Venn diagram to show the low-risk group for lymph node metastasis and how they satisfied the various guidelines (Figure 1). In our cohort, candidate fertility-sparing therapies were the most conservative for identifying a group at low risk for lymph node metastasis, and patients designated as being at low risk, based on fertility-sparing therapy criteria, also completely satisfied KSGO criteria. By using SOGC and INCa guidelines, we would have identified more low-risk group patients than by using the KSGO guidelines, without a significant increase in the false-negative rate.

## DISCUSSION

In this study, we evaluated the role of preoperative assessment based on MRI and histological analysis in identifying a group at low-risk of endometrial cancer. When the grades or MR stages are considered separately, the overall agreement between preoperative and postoperative findings was poor. However, a combined analysis of preoperative parameters is highly sensitive in predicting extra-uterine disease, in particular lymph node metastasis, and may result in the accurate identification of patients with endometrial cancer who are at low risk for lymph node metastasis.

The first objective of this study was to correlate the grade and stage based on preoperative assessment with that of the final pathology. Our study results correlated with those of previous studies [13–15]. Body et al. showed that preoperative assessment had a 90% negative-predictive value for the diagnosis of grade 3 tumors [15]. Batista et al. suggested that preoperative endometrial sampling was found to be a modest overall predictor of postoperative histological grading [14]. They showed that 15.2% of patients with preoperative grade 1 diagnosis were upgraded after hysterectomy. Baek et al. found extensive upgrading (16.2%), based on the final pathology report from hysterectomy specimens in cases of preoperative grade 1 disease [13]. In line with previous reports, the overall level of agreement between preoperative and postoperative grading was also disappointing. We showed that upgrading occurred in 19.7% of patients with preoperative grade 1 disease. In addition, previous studies have demonstrated the diagnostic performance of MRI for evaluating myometrial invasion and lymph node metastasis [15–17]. Some studies have specifically evaluated the performance of MRI for FIGO staging and have shown an underestimation rate of approximately 20% [15]. This finding is similar to that of our study; thus, preoperative evaluation based on MRI is incomplete.

Current guidelines recommend a more selective and tailored lymphadenectomy rather than routine lymphadenectomy in the management of endometrial cancer. The NCCN guidelines suggest that the extent of surgical staging can be determined based on preoperative and intraoperative findings [4]. Deciding whether to perform lymphadenectomy based on preoperative findings has some advantages. As there has been increasing demand for surgeons to provide the necessary information prior to treatment, this is suitable for preoperative decision counseling. However, there is currently no consensus definition of a low-risk group for lymph node metastasis. Several guidelines have suggested that, for patients meeting the low-risk criteria, lymphadenectomy could be omitted safely, based on preoperative parameters [5, 7, 8]. Considering that few studies have validated the characteristics of low-risk criteria used in the current guidelines, our study has value in that it evaluated the performance of the preoperative criteria used in the current guidelines, and simultaneously compared them in an independent cohort.

We showed that the low-risk group as defined by any of the guidelines had an acceptable rate of lymph node metastasis, and a high negative-predictive value. This suggests that the decision to omit lymphadenectomy based on any of the guidelines could be justified. A previous study has shown treatment preferences for no lymphadenectomy over routine lymphadenectomy in early-stage endometrial cancer [18]. They showed that the majority of patients would accept a small amount of recurrence risk (within 3%) to reduce the incidence of lymphedema from routine lymphadenectomy. Under a selective lymphadenectomy strategy, patients who were unstaged based on false-negative results from low-risk group are likely to have a higher recurrence risk due to unidentified metastatic disease. In our study, the false-negative rate from models did not exceed 3%.

In our cohort, fertility-sparing therapies identified the low-risk group very conservatively, while the Canadian and French guidelines identified more candidates for such therapies, omitting lymphadenectomy, with a low likelihood of lymph node metastasis. Our study demonstrates that the current guidelines may be considered safe for practice.

Decisions regarding fertility-sparing therapies are solely dependent on clinical staging based on a preoperative assessment. In Korea, approximately 11.5% of endometrial cancer patients were younger than 40 years, which is higher than the 4% of such patients in the US [19, 20]. Candidates for fertility-sparing therapies are generally considered to be women younger than 40 years, with the disease limited to the endometrium, well-differentiated, endometrioid, with no extra-uterine disease, and who have a strong desire to preserve the uterus. We have shown that the risk of extra-uterine disease is low in this disease subset. In addition, there is little evidence that fertility-sparing therapies could be considered for reproductive-aged patients who have grade 2, stage IA endometrioid adenocarcinoma with myometrial invasion. Given the efforts to expand the number of candidates eligible for fertility-sparing therapies, KGOG-2020 is a single-arm phase II study evaluating the efficacy of fertility-sparing management in young women with stage I endometrial cancer with grade 2 or superficial myometrial invasion. In our study, when expanding fertility-sparing therapies in KGOG-2020, the rate of extra-uterine disease was low, and the criteria used had a high negative-predictive value for lymph node metastasis.

In conclusion, the criteria used in the current guidelines can identify a group with a low risk of lymph node metastasis. This study has useful implications for physicians counseling patients who desire to avoid lymphadenectomy. Our findings suggest that the vast majority of women could safely avoid lymphadenectomy, with its potential increased lymphedema risk.

## MATERIALS AND METHODS

From January 2012 to December 2014, 537 patients with endometrial cancer were enrolled into a prospective, multicenter cohort study (KGOG-2015), involving 20 hospitals in three countries, i.e., Korea, Japan, and China [10]. All participating patients had biopsy-confirmed endometrial cancer and underwent preoperative MRI and CA-125 before surgery. Comprehensive surgical staging, including systematic lymphadenectomy, was performed for all patients in this study. Patients were staged on the basis of final pathological findings according to the 2009 FIGO classification. Nine patients were excluded due to inadequate imaging study (n = 1), no lymph node dissection (n = 4), and proven sarcoma (n = 3). All patients underwent MRI of the pelvis at either 1.5T or 3T scanners with a phased-array body coil. MR sequences of each institution varied because of our retrospective study design, but the common sequences in all institutions were precontrast T2-weighted images, precontrast T1-weighted images (T1W1), post-contrast T1W1 after a standard dose of gadolinium administration, and diffusion weighted image. There were no sequences with slice thickness larger than 7.5mm.

### Data collection

After gaining approval from the Institutional Review Board of Yonsei University Hospital, the following data were collected for all patients: patient characteristics (age, body mass index [BMI], menopausal status), preoperative assessment (MRI finding, histological results, CA-125), information about surgery (type of procedure, approach, extent of surgery), postoperative pathological findings.

For preoperative MRI, the data for preoperative assessment (myometrial invasion depth, tumor size, suspicious lymph node metastasis, cervical, adnexa, and serosal involvement, vaginal involvement, extra-uterine metastasis) were collected. For preoperative histology, tumor grade, histological subtypes, the methods used (dilatation & curettage, endometrial sampling, hysteroscopy) were recorded. In terms of postoperative pathological findings, histological subtypes, tumor grade, cytological results, lymphovascular space invasion, and FIGO stage 2009 were recorded. No central review was performed for preoperative MRI and pathologic examinations, and details of measurements have been described in a previous report [10].

### Definition of a low-risk group

Three models based on preoperative assessment were identified from guidelines via a PubMed search. The Korean Society of Gynecologic Oncology (KSGO) defines the low-risk group for lymph node metastasis as preoperative grade 1, with endometrioid histology and superficial myometrial involvement [5, 12]. The Society of Obstetricians and Gynecologists of Canada (SOGC) defines low-risk disease as grade 1 adenocarcinoma on preoperative biopsy, and suggests that lymphadenectomy could be omitted in this low-risk group [7]. The French National Cancer Institute (INCa) classifies three risk groups, based on preoperative assessment, as follows: low risk—stage IA, grades 1 or 2, endometrioid type; intermediate risk—stage IA, grade 3, and stage IB, grades 1 or 2, endometrioid type; high risk—stage IB, grade 3, endometrioid type, and any stage with non-endometrioid type [8]. They do not recommend lymphadenectomy in the low-risk group.

In terms of fertility-sparing therapies, the current guidelines suggest that this option should be selectively used for young women who desire fertility preservation. The NCCN and KSGO guidelines recommend fertility-sparing therapies in selected patients with biopsy-proven grade 1, stage IA (limited to the endometrium on MRI) endometrioid adenocarcinoma [4, 12]. Decisions regarding fertility-sparing therapies are solely dependent on preoperative assessment. The KGOG-2020 is a single-arm phase II trial evaluating the feasibility and safety of expanding fertility-sparing therapies to stage IA (superficially invading myometrium) and grade 2.

### Statistical analysis

The results of the preoperative assessment were compared with the final pathological analysis of the surgical specimen. In terms of tumor grade, the agreement levels between the preoperative and postoperative pathological findings were analyzed using Kappa statistics with 95% confidence intervals. Descriptive analysis was performed to evaluate the rate of upstaging during surgical staging, as compared to preoperative MRI staging. In addition, we evaluated the performance of preoperative low-risk criteria, including those of the KSGO, SOGC, INCa, and fertility-sparing therapies. The area under the curve (AUC) was calculated for predicting lymph node metastasis. Analysis was performed using STATA 12.0 (StatCorp, College Station, TX, USA). P < 0.05 was considered to be statistically significant and all P-values were two-sided. Patients with missing or unclear data for calculating the probability of lymph node metastasis were excluded when evaluating each criterion. No imputation method was applied.
